# Bloodstream infections at a tertiary level paediatric hospital in South Africa

**DOI:** 10.1186/s12879-017-2862-2

**Published:** 2017-12-06

**Authors:** Harsha Lochan, Vashini Pillay, Colleen Bamford, James Nuttall, Brian Eley

**Affiliations:** 1 Paediatric Infectious Diseases Unit, Red Cross War Memorial Children’s Hospital and the Department of Paediatrics and Child Health, University of Cape Town, Cape Town, South Africa; 2 Division of Medical Microbiology, University of Cape Town and the National Health Laboratory Service, Groote Schuur Hospital, Cape Town, South Africa

**Keywords:** Bloodstream infections, Children, Africa, Antimicrobial resistance

## Abstract

**Background:**

Bloodstream infection (BSI) in children causes significant morbidity and mortality. There are few studies describing the epidemiology of BSI in South African children.

**Methods:**

A retrospective descriptive cohort study was conducted at a paediatric referral hospital in Cape Town, South Africa. The National Health Laboratory Service (NHLS) microbiology database was accessed to identify positive blood culture specimens during the period 2011–2012. Demographic and clinical details, antimicrobial management and patient outcome information were extracted from medical and laboratory records. Antibiotic susceptibility results of identified organisms were obtained from the NHLS database.

**Results:**

Of the 693 unique bacterial and fungal BSI episodes identified during the study period, 248 (35.8%) were community-acquired (CA), 371 (53.5%) hospital-acquired (HA) and 74 (10.7%) healthcare-associated (HCA). The overall risk was 6.7 BSI episodes per 1000 admissions. *Escherichia coli, Staphylococcus aureus* and *Streptococcus pneumoniae* were the most frequent causes of CA-BSI and *Klebsiella pneumoniae, Acinetobacter baumanii* and *S.aureus* were most commonly isolated in HA-BSI. On multivariable analysis, severe underweight, severe anaemia at the time of BSI, admission in the ICU at the time of BSI, and requiring ICU admission after BSI was diagnosed were significantly associated with 14-day mortality.

**Conclusion:**

This study adds to the limited literature describing BSI in children in Africa. Further studies are required to understand the impact that BSI has on the paediatric population in sub-Saharan Africa.

## Background

Bloodstream infection (BSI) causes significant morbidity and mortality in children and increase healthcare expenditure [1, 2]. In Africa, community-acquired (CA)-BSI was identified in 5.8% of children presenting to hospital in Tanzania and up to 19.9% in rural Ghana [3, 4], while hospital-acquired (HA) BSI was less frequently described. In a recent study among children hospitalised in a Kenyan district hospital, the overall risk of acquiring a nosocomial BSI was 5.9 per 1000 admissions. [5]. South Africa has a paucity of literature describing BSI in children. In one study, the risk of CA-BSI and HA-BSI was 16 and 5 per 1000 admissions, respectively [6]. More recent studies have documented lower pathogen yields and high contamination rates in blood culture specimens. The spectrum of pathogens described was similar to elsewhere in Africa with the exception of lower rates of infection caused by non-typhoidal *Salmonella species* [7, 8].

Clinical manifestations of childhood BSI in sub-Saharan Africa (SSA) are also poorly documented. Fever may be the only manifestation of childhood invasive infection. In SSA with the added burden of malaria, HIV-infection, tuberculosis and malnutrition, the treatment of fever is often syndromic, targeting multiple possible aetiologies, without necessarily considering BSI as a potential cause [9]. Not all fever is due to malaria, as shown in a recent Tanzanian study where viruses and bacteria caused more than 90% of childhood fevers. In that study only 6.4% of fever episodes were caused by malaria [10]. In HIV-infected children treated with antiretroviral therapy (ART), the risk of BSI is highest in the first 3 months after ART initiation with a steady decline thereafter [11].

Antimicrobial resistance is an important global public health threat. A systematic review on antimicrobial resistance among gram-negative bacteria in developing countries including 15 studies from Africa showed that in children, 50% and 11% of *Escherichia coli* isolates were resistant to ampicillin and gentamicin, respectively and 30% of *Klebsiella pneumoniae* isolates were resistant to ceftriaxone [12]. These antimicrobials are used empirically to treat ill children with invasive infection. None of the studies in this review included children from South Africa but Dramowski and colleagues recently showed that more than 75% of *K. pneumoniae* isolates at their hospital in Cape Town were extended spectrum beta-lactamase (ESBL) producing organisms [7].

Despite the growing body of literature there remains many gaps in our knowledge about the clinical and laboratory aspects of BSI in SSA. To address some of these aspects of BSI in SSA we conducted an observational study aimed at describing the epidemiology, clinical manifestations and antimicrobial management of BSI at Red Cross War Memorial Children’s Hospital (RCWMCH).

## Methods

### Study design and setting

This retrospective descriptive cohort study was completed at RCWMCH on children with culture-confirmed BSI, diagnosed between 1 January 2011 and 31 December 2012. RCWMCH is a 273-bed tertiary level paediatric hospital in Cape Town, Western Cape province, South Africa. The hospital has emergency, general paediatric, specialised paediatric, surgical, burns, trauma and intensive care facilities serving as a referral centre for the Western Cape and surrounding provinces. Dedicated early neonatal care linked to obstetric services is provided by other hospitals in Cape Town. However, neonates requiring immediate surgical care, or medical care after the first 7 days of life are admitted to RCWMCH.

### Study population

**Fig. 1:**
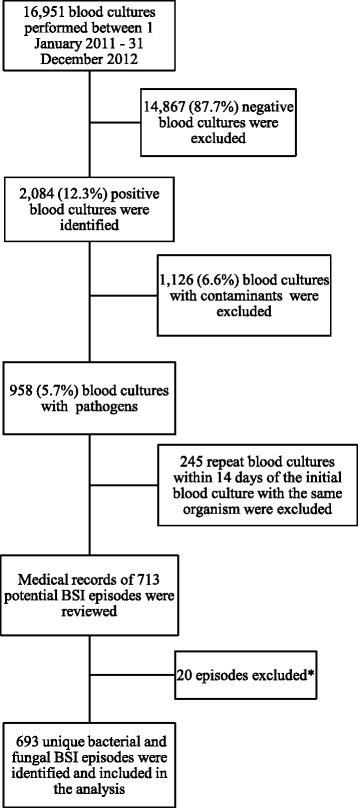
Selection of BSI episodes for data analysis at Red Cross War Memorial Children’s Hospital (RCWMCH). ^*^14 post-mortem specimens and 6 non-blood specimens (peritoneal fluid [3], eye fluid aspirate [1], pus [1] and lymph node aspirate [1]) inoculated in blood culture bottles

Most children hospitalised in RCWMCH originate from poor, peri-urban communities in the Western Cape. During the study period there were 43,663 admissions to the hospital and 16,951 blood culture specimens were processed from patients with suspected invasive infection, i.e. 1 blood culture specimen per 2.6 hospital admissions. The medical microbiology database of the National Health Laboratory Service (NHLS) was accessed, and 2084 positive blood culture results of children hospitalised at RCWMCH during the study period were identified. Organisms isolated from these blood cultures were stratified into pathogens and non-pathogens. Coagulase-negative staphylococci, *Staphylococcus epidermidis*, *Bacillus* spp*., Micrococcus* spp*.,* viridans streptococci, and coryneform bacteria were regarded as contaminants and excluded from the analysis unless they were isolated in two or more independent blood culture specimens from the same patient within a 48-h period, in which case they were included as a pathogen [13]. Within this group of blood culture results, repeat blood cultures within 14 days of the initial blood culture and yielding an identical pathogen were regarded as part of the same potential BSI episode, and therefore excluded. A recurrent BSI episode was defined as the isolation of the same or different organism on blood culture more than 14 days after the initial/previous BSI episode. To verify each BSI episode (new or recurrent) identified during this laboratory review, the medical records of all patients linked to these events were reviewed. During this process 20 episodes were excluded as they had been misclassified as BSI episodes. Thus the study population comprised 548 children who experienced 693 unique bacterial or fungal BSI episodes (Fig. 1).

### Data collection

Paper-based medical records of patients with BSI episodes were reviewed and relevant data was extracted and manually transferred onto standardised data collection forms. HIV status was determined using HIV test results obtained from patient medical and laboratory records. Microbiological results obtained from the NHLS database were also entered on the data collection forms.

### Microbiological procedures

All microbiology testing was conducted at the Groote Schuur National Health Laboratory Service (NHLS) microbiology laboratory. The laboratory uses the BACTEC™ 9240 automated blood culture system (Becton Dickinson, Sparks, Maryland). For blood culture bottles flagging positive with mono-morphic Gram-negative bacilli seen on Gram stain, a locally validated method of direct inoculation into the automated Vitek®2 system (bioMérieux, Inc., France) was employed, using Vitek®2 ID-GNB and AST-N133 cards for identification and susceptibility testing respectively. This direct method was supplemented, where necessary with repeat testing from bacterial colonies subcultured onto agar plates, using either Vitek®2, disk diffusion or gradient diffusion E-test (bioMérieux, Marcy l’Etoile, France) methods. Standard biochemical methods and disk diffusion and gradient diffusion antibiotic susceptibility tests were used to evaluate Gram-positive organisms, while yeasts were tested with the Vitek®2’s YST identification and AST-YS07 cards.

Susceptibility results were interpreted according to the Clinical Laboratory Standards Institute (CLSI) criteria for 2011 and 2012 [14, 15]. Ertapenem minimum inhibitory concentration (MIC) breakpoints for Gram-negative organisms were revised in 2012, as were the ciprofloxacin breakpoints for *Salmonella* species. For *Pseudomonas* species, the breakpoints for meropenem, imipenem and piperacillin-tazobactam were revised in 2012.

ESBLs were detected by the Vitek®2 Advanced Expert system or by the double disk synergy method if using disk diffusion testing. Despite the changes to reporting of cephalosporin susceptibility in ESBL-producing organisms introduced by CLSI in 2010, the laboratory, in line with the contemporary national practice, continued to report ESBL- producing *Enterobacteriaceae* as resistant to all cephalosporins.

### Study definitions

BSI was classified as (1) *community-acquired* (CA) if the pathogen was isolated within the first 48 h after admission and did not meet the definition of healthcare-associated infection, (2) *hospital-acquired* (HA) if the pathogen was isolated from a blood culture obtained more than 48 h after hospitalisation without the patient having been a resident of a long-term care facility or hospitalised during the preceding 28 days, or (3) *healthcare-associated* (HCA) if the pathogen was isolated in a patient within the first 48 h of being hospitalised from a long-term care facility, or readmitted within 28 days of discharge from a hospital or birth facility [5, 13]. BSI with a clinical focus refers to a laboratory confirmed BSI event accompanied by one of more clinical sites of infection such as pneumonia, meningitis, soft tissue infection or line infection as extracted from the patient’s medical records.

The HIV status was defined as follows: (1) *HIV-infected*: a child < 18 months old with a positive HIV DNA PCR result confirmed by either a quantitative HIV RNA PCR or repeat HIV DNA PCR test, or a child ≥ 18 months old with 2 positive serological test results (HIV ELISA or HIV Rapid test) or a positive HIV DNA PCR result confirmed by either a quantitative HIV RNA PCR or repeat HIV DNA PCR test, (2) *HIV-uninfected*: a child with a negative HIV serological or HIV DNA PCR result and (3) *Unknown HIV status:* a child with unknown maternal HIV status and who was not tested for HIV infection.

Moderate and severe underweight for age (UWFA) were defined as weight-for-age z score (WAZ) between −2 and −3 standard deviations (SD) below the median World Health Organisation (WHO) growth reference standards, and a WAZ < −3 SD respectively [16].

Antimicrobial management at the time of the BSI was considered to be effective if the organism isolated was susceptible to the prescribed antimicrobial agent.

### Statistical analysis

All data was entered anonymously into an Excel spreadsheet and analysed using Stata release 12.0 statistical software package (STATACorp, College Station, Texas). Continuous variables were expressed as medians (interquartile range, IQR). Frequencies and proportions were used to describe categorical variables. Chi-squared and Kruskal-Wallis tests of association were used to assess associations between categorical and numerical variables across the type of BSI for the baseline patient characteristics and BSI with or without a clinical focus for blood marker variables. Cox proportional hazards regression was used to assess factors associated with 14-day mortality, adjusting for the baseline demographic, clinical and blood marker variables. *P*-values < 0.05 were considered significant.

## Results

### Classification and risk of bloodstream infection

Overall, 248 (35.8%) CA-, 371 (53.5%) HA- and 74 (10.7%) HCA-BSI episodes were identified. Fewer blood cultures were processed and fewer BSI episodes occurred in 2012 compared to 2011. The overall risk of CA-BSI, HA-BSI and HCA-BSI was 5.7, 8.5 and 1.7 episodes per 1000 admissions, respectively (Table 1).

**Table 1:** Classification and risk of bloodstream infection (BSI), 2011–2012

	2011	2012	2011–2012
Hospital admissions	22,685	20,978	43,663
Blood cultures processed	9108	7843	16,951
Classification of bloodstream infection (n/N(%))			
Community-acquired	151/413 (36.6)	97/280 (34.6)	248/693 (35.8)
Hospital-acquired	214/413 (51.8)	157/280 (56.1)	371/693 (53.5)
Healthcare-associated	48/413 (11.6)	26/280 (9.3)	74/693 (10.7)
Risk of BSI per 1000 hospital admissions			
Community-acquired	6.7	4.6	5.7
Hospital-acquired	9.4	7.5	8.5
Healthcare-associated	2.1	1.2	1.7

### Baseline characteristics of study participants

Table 2 describes the baseline characteristics of the study participants at the time of each BSI episode and disaggregates the information according to the type of BSI. The median age at the time of BSI was 11.5 months (IQR 3.6–50). Furthermore, 40.7% (273/670) of episodes occurred in children who were moderately or severely underweight. Of the 524 children (75.6%) whose HIV status was known at the BSI event, 13.4% (70/524) were HIV-infected.

**Table 2:** Baseline patient characteristics

	CA-BSI (*N* = 248^a^)	HA-BSI (*N* = 371^a^)	HCA-BSI (*N* = 74)	Total BSIs (*N* = 693^a^)
Age median (IQR)	9.3 (3.1–39.3)	13.0 (4.0–59.7)	7.1 (1.4–67.0)	11.5 (3.6–50.0)
Age categories, n/N(%)				
0–28 days	31 (12.5)	28 (7.5)	16 (21.6)	75 (10.8)
1 - < 2 months	17 (6.9)	17 (4.6)	6 (8.1)	40 (5.8)
2 - < 12 months	88 (35.5)	132 (35.6)	22 (29.7)	242 (34.9)
12 months – < 5 years	67 (27)	106 (28.6)	11 (14.9)	184 (26.5)
> 5 years	45 (18.1)	88 (23.7)	19 (25.7)	152 (21.9)
WAZ median (IQR)	−1.2 (−2.5–0.1)	−1.8 (−3.4–0.6)	−1.9 (−3.3–1.0)	−1.6 (−3–0.3)
WAZ categories, n/N (%)				
Severe underweight	44/240 (18.3)	103/361 (28.5)	22/69 (31.9)	169/670 (25.2)
Moderate underweight	33/240 (13.8)	60/361 (16.6)	11/69 (15.9)	104/670 (15.5)
Mild-normal	163,240 (67.9)	198/361 (54.8)	36/69 (52.2)	397/670 (59.3)
HIV-infected, n/N (%)	33 (13.3)	33 (8.9)	4 (5.4)	70 (10.1)
HIV-uninfected, n/N (%)	172 (69.3)	232 (62.5)	49 (66.2)	454 (65.5)
HIV not tested, n/N (%)	43 (6.2)	106 (28.6)	21 (28.4)	169 (24.4)
Receiving ART at the time of the BSI in HIV infected child, n/N(%)	11/33 (33.3)	29/33 (87.9)	3/4 (75)	43/70 (61.4)

^a^ *N* denominator used unless otherwise stated, *IQR* interquartertile range, *BSI* bloodstream infection, *CA-BSI* community-acquired bloodstream infection, *HA-BSI* hospital-acquired bloodstream infection, *HCA-BSI* healthcare-associated bloodstream infection, *WAZ* weight-for-age z-score, *HIV*-*infected* human immunodeficiency virus infected, *ART* antiretroviral therapy

### Clinical spectrum of bloodstream infections

A clinical site of infection in addition to the bloodstream was identified in 481/663 (72.5%) of the BSI episodes, of which 327/481(68.0%) had a single identifiable site of infection. Two or more clinical sites of infection were identified in the remaining 154 BSI episodes (Table 3). Pneumonia and gastroenteritis were commonly diagnosed at the time of the BSI episode. There was no significant difference in the proportions of BSI episodes with and without a clinical focus associated with a temperature ≥ 38° C; 60% (270/450) vs 62.8% (108/172), RR = 0.99 (95% CI: 0.86–1.14).

**Table 3:** Clinical classification of bloodstream infection (BSI)

	CA-BSI (*N* = 242^a^)	HA-BSI (*N* = 353^a^)	HCA-BSI (*N* = 68^a^)	Total BSIs (*N* = 663^a^)
	n/N (%))	n/N (%)	n/N (%)	n/N (%)
BSI with no clinical focus	28 (11.6)	131(37.1)	23 (33.8)	182 (27.5)
Hypothermia^b^	5/28 (17.9)	2/131 (1.5)	0/23 (0)	7/182 (3.8)
Fever^c^	14/28 (50)	77/131 (58.8)	17/23 (74)	108/182 (59.3)
BSI with a clinical focus	214(88.4)	222(62.9)	45(66.2)	481 (72.5)
Hypothermia^b^	11/214 (5.1)	4/222 (1.8)	5/45 (11.1)	20/481 (4.2)
Fever^c^	112/214 (52.3)	136/222 (61.3)	22/45 (48.9)	270/481 (56.1)
Pneumonia	65/214 (30.4)	91/222 (41)	16/45 (35.6)	172/481 (35.8)
Gastroenteritis	67/214 (31.3)	28/222 (12.6)	10/45 (22.2)	105/481 (21.8)
Soft tissue infection^d^	18/214 (8.4)	50/222 (22.5)	4/45 (8.9)	72/481 (15)
Meningitis	39/214 (18.2)	1/222 (0.5)	13/45 (28.9)	53/481 (11)
UTI	29/214 (13.6)	20/222 (9)	4/45 (8.9)	53/481 (11)
Line sepsis	4/214 (1.9)	29/222 (13.1)	3/45 (6.7)	36/481 (7.4)
URTI	20/214 (9.3)	8/222 (3.6)	3/45 (6.7)	31/481 (6.4)
Empyema	4/214 (1.9)	5/222 (2.3)	–	9/481 (1.9)
Septic arthritis	6/214 (2.8)	3/222 (1.4)	–	9/481 (1.9)
Bronchiolitis	4/214 (1.9)	–	1/45 (2.2)	5/481 (1)
Pleural effusion	–	3/222 (1.4)	–	3/481 (0.6)

*BSI* bloodstream infection, *CA-BSI* community-acquired bloodstream infection, *HA-BSI* hospital-acquired bloodstream infection, *HCA-BSI* healthcare-associated bloodstream infection, ^a^N = denominator used unless otherwise stated; ^b^Body temperature ≤ 35.5 ° C around the time of BSI; ^c^Body temperature ≥ 38 °C around the time of BSI; ^d^Soft tissue infection includes skin abscesses, burn wound infections, wound sepsis and infected eczema or scabies, *UTI* urinary tract infection, *URTI* upper respiratory tract infection

### Spectrum of organisms

Fifty two different organisms were isolated from the 693 BSI episodes including 11 fungi. Fifty eight percent of the BSI episodes were caused by gram-negative bacteria, 36% by gram-positive bacteria and 6% by fungi. *Candida albicans* (19/42) was the dominant fungal isolate. *Cryptococcus neoformans* was isolated in one HIV-infected child with severe immunodeficiency i.e. an absolute CD4 count of < 20 × 10^9^/L and a CD4 percentage of < 1% (Table 4).

**Table 4:** Spectrum of organisms identified during the study period 2011–2012

	CA-BSI n/N (%)	HA-BSI n/N (%)	HCA-BSI n/N (%)	Total BSIs n/N (%)
	(*N* = 248)	(*N* = 371)	(*N* = 74)	(*N* = 693)
Gram-negative bacteria	117 (47.2)	240 (64.7)	43 (58.1)	400 (57.7)
* Escherichia coli*	49 (19.8)	33 (8.9)	10 (13.5)	92 (13.3)
* Klebsiella pneumoniae*	5 (2)	78 (21)	9 (12.2)	92 (13.3)
* Acinetobacter baumanii*	4 (1.6)	54 (14.6)	1 (1.4)	59 (8.5)
* Pseudomonas aeruginosa*	3 (1.2)	19 (5.1)	4 (5.4)	26 (3.8)
Non-typhoidal *Salmonella*	11 (4.4)	6 (1.6)	3 (4.1)	20 (2.9)
* Enterobacter cloacae*	3 (1.2)	12 (3.2)	3 (4.1)	18 (2.6)
* Serratia marcescens*	0 (0)	17 (4.6)	1 (1.4)	18 (2.6)
* Neisseria meningitidis*	12 (4.8)	0 (0)	1 (1.4)	13 (1.9)
* Haemophilus influenza B*	6 (2.4)	1 (0.3)	2 (2.7)	9 (1.3)
* Salmonella typhi*	4 (1.6)	0 (0)	0 (0)	4 (0.6)
Other	20 (8.1)	20 (5.4)	9 (12.2)	49 (7.1)
Gram-positive bacteria	124 (50)	97 (26.1)	28 (37.8)	249 (35.9)
*Staphylococcus aureus*	55 (22.2)	37 (10)	11 (14.9)	103 (14.9)
*Streptococcus pneumoniae*	45 (18.1)	6 (1.6)	6 (8.1)	57 (8.2)
*Enterococcus faecalis*	4 (1.6)	23 (6.2)	5 (6.8)	32 (4.6)
*Streptococcus group B*	13 (5.2)	0 (0)	4 (5.4)	17 (2.5)
*Enterococcus faecium*	2 (0.8)	14 (3.8)	0 (0)	16 (2.3)
Other	5 (2)	17 (4.6)	2 (2.7)	24 (3.5)
Mycobacterium tuberculosis	1 (0.4)	1 (0.3)	0 (0)	2 (0.3)
Fungi	6 (2.4)	33 (8.9)	3 (4.1)	42 (6.1)
* Candida albicans*	1 (0.4)	16 (4.3)	2 (2.7)	19 (2.7)
* Candida parapsilosis*	1 (0.4)	9 (2.4)	1 (1.4)	11 (1.6)
Other *Candida* species	4 (1.6)	8 (2.2)	0 (0)	12 (1.7)

N = denominator used unless otherwise stated

### Susceptibility to antimicrobial agents

The majority of *Staphylococcus aureus* isolates 81/103 (78.6%) were susceptible to cloxacillin. The remaining 22 isolates were methicillin-resistant of which 17 were HA- BSI, accounting for 17/371 (5%) of all HA-BSI. Of the 57 *S. pneumoniae* isolates, 26.3% (15/57) were resistant to penicillin according to meningitis criteria with MIC > 0.06 μg/ml. Susceptibility testing for ceftriaxone and vancomycin was conducted in 52 and 21of the *S. pneumoniae* isolates, respectively with100% susceptibility for both antimicrobials. Of the 48 *Enterococcus* isolates, 54.2% (26/48) were susceptible to ampicillin and all were susceptible to vancomycin.

Table 5 describes antimicrobial susceptibility of the most commonly isolated Gram-negative organisms. Only 9.9% (9/91) of *E.coli* isolates were susceptible to ampicillin while 70.3% (64/91) were susceptible to gentamicin. Thirty four percent (31/92) of all *E.coli* isolates were extended-spectrum beta lactamase (ESBL)-producing organisms of which 10 and 16 were community-acquired and hospital-acquired respectively, accounting for 4% (10/248) and 4.3% (16/371) of CA- and HA-BSI, respectively. By contrast, 77.3% (68/88) of *K. pneumoniae* isolates were ESBL producers which accounted for 16.7% (62/371) of HA-BSI. No carbapenem-resistant *Enterobacteriacae* (CRE*)* isolates were identified on blood culture during the study period. Resistance to both aminoglycosides and carbapenems was present in 22% (13/59) of *A. baumanii* isolates. All *A. baumanii* isolates were susceptible to colistin. All non-typhoidal *Salmonella ssp.* isolates were susceptible to ceftriaxone, with only a single isolate not susceptible to ampicillin and ciprofloxacin. *Candida albicans* and *parapsilosis* isolates showed a 100% susceptibility to fluconazole.

**Table 5:** Rates of antimicrobial susceptibility for the common gram-negative organisms identified

	*Acinetobacter baumanii* (*N* = 59)		*Escherichia coli* (*N* = 92)		*Klebsiella pneumoniae* (*N* = 92)		*Pseudomonas aeruginosa* (*N* = 26)		Non-typhoidal *Salmonella* (*N* = 20)	
Antimicrobial	No isolates tested	No (%) Susceptible	No isolates tested	No (%) Susceptible	No isolates tested	*N* (%) Susceptible	No isolates tested	N (%) Susceptible	No isolates tested	*N* (%) Susceptible
Ampicillin	–	–	91	9 (9.9)	92	–	–	–	20	19 (95)
Co-amoxyclav^a^	–	–	89	43 (48.3)	91	27 (29.7)	–	–	1	1 (100)
Ceftriaxone	–	–	90	59 (65.6)	88	20 (22.7)	–	–	20	20 (100)
Ceftazidime	59	24 (40.7)	91	60 (65.9)	91	20 (22)	26	23 (88.5)	1	1 (100)
Ciprofloxacin	59	45 (76.3)	87	70 (80.5)	85	52 (61.2)	26	15 (57.7)	19	18 (94.7)
Piperacillin-tazobactam	58	10 (17.2)	6^$^	5 (83.3)	49^$^	34 (69.4)	7^$^	1 (14.3)	1^$^	1 (100)
Gentamicin	59	27 (45.8)	91	64 (70.3)	89	25 (28.1)	25	15 (60)	–	–
Amikacin	58	26 (44.8)	92	77 (83.7)	91	70 (76.9)	26	16 (61.5)	–	–
Meropenem	56	26 (46.4)	92	92 (100)	92	92 (100)	26	22 (84.6)	20	20 (100)
Imipenem	49	27 (55.1)	90	90 (100)	90	90 (100)	26	21 (80.8)	20	20 (100)
Ertapenem			92	92 (100)	91	91 (100)	–	–	20	20 (100)
Colistin	59	59 (100)	90	90 (100)	89	89 (100)	26	23 (88.5)	–	–

^a^Co-amoxyclav = Amoxycillin-clavulanic acid co-formulation $ due to technical limitations with the Vitek®2 AST card piperacillin-tazobactam was infrequently reported in 2011–2012; *No* number

### Antimicrobial therapy

Information on antimicrobial therapy was available for 92% (638/693) of BSI episodes. The median (IQR) time to effective antibiotic therapy for bacterial isolates of CA-, HA- and HCA- BSI was 0 (0–1) day, 1 (0–2) day and 1 (0–3) day, respectively. The median (IQR) time to effective antifungal therapy for fungal isolates was 4.5 (4–5) days, 3 (0.5–4) days and 2.5 (2–3) days for CA-, HA- and HCA-BSI, respectively. Of the 638 episodes, 592 (92.8%) `received effective antimicrobial therapy based on the susceptibility profile of the isolate. In the remaining 46 episodes, 42 were treated with an ineffective antimicrobial, and 4 fungal BSI episodes did not receive antifungal therapy. Of the 42 bacterial episodes not treated with an effective antimicrobial, 10 (23.8%) resulted in a fatal outcome with the median (IQR) time to death of 4 (2–9) days, and the remaining 32 (76.2%) episodes resolved on ineffective antibiotic therapy. In 2 of the 4 fungal episodes that were not treated with antifungal therapy the patients died before the culture results were known. The remaining 2 children recovered uneventfully.

### Outcomes

85 % (590/693) of BSI episodes were successfully treated and the children were discharged from hospital after these episodes. During the study period, 103 (14.9%) children died during or after a BSI episode prior to hospital discharge. Seventy of these deaths occurred within 14 days as a direct result of the BSI and were included in the survival analysis as there was a high chance of correctly identifying relevant risk factors in these children. The median time (IQR) to death of these 70 children was 3 (1–8) days. Of the 33 deaths that occurred after 14 days, the median time (IQR) to death was 46 (23–67) days.

Table 6 describes risk factors associated with 14-day inpatient mortality in children with BSI. On multivariable analysis, severe UWFA, severe anaemia at the time of BSI, admission in the ICU at the time of BSI, and requiring ICU admission after BSI was diagnosed were significantly associated with 14-day mortality. HIV-infection was not associated with increased mortality risk.

**Table 6:** Risk factors associated with 14-day inpatient mortality in children with BSI

Factor	Univariate HR (95% CI)	*P* value	Adjusted HR (95%CI)	*P* value
Age	*N* = 693			
0–28 days	0.9 (0.4–2)	0.834	1	
1 - < 2 months	2.2 (1.1–4.6)	0.035	1	
2 - < 12 months	1.2 (0.8–2)	0.376	1	
12 months – 5 years	0.7 (0.4–1.2)	0.219	1	
> 5 years	1		1	
Nutritional status	*N* = 693			
Severe underweight for age	2.4 (1.5–3.9)	0.0001	2.0 (1.2–3.3)	0.007
Moderate underweight for age	1.1 (0.6–2)	0.851	1	
Mild-normal weight for age	1		1	
Weight not recorded	2.4 (1–5.9)	0.060	4.9 (1.2–20.8)	0.029
Haemoglobin	*N* = 693			
Severe anaemia (< 7 g/dL)	3.3 (1.7–6.4)	0.001	2.7 (1.3–5.6)	0.006
Mild anaemia (7–11 g/dL)	1 (0.6–1.7)	0.909	1	
No anaemia (≥ 11 g/dL)	1		1	
Result not available	3.7 (1.2–11.9)	0.025	4.2 (1.3–13.7)	0.018
ICU management	*N* = 693			
ICU resident at time of BSI	1.5 (0.89–2.7)	0.124	6.5 (3.7–11.6)	0.0001
BSI requiring ICU management	5.2 (3.3–8.4)	0.0001	3.5 (1.8–6.7)	0.0001
BSI not requiring ICU management	1		1	
Type of BSI	*N* = 693			
CA	1		1	
HA	1.2 (0.8–2)	0.377	1	
HCA	0.9 (0.4–1.9)	0.806	1	
HIV infection	*N* = 693			
Yes	1.5 (0.8–2.9)	0.221	1	
No	1		1	
Unknown status	0.8 (0.5–1.5)	0.523	1	

## Discussion

The current study describes the aetiology and quantifies the risk of BSI at our hospital. Gram-negative organisms were the predominant cause of BSI. The risk was lower for CA-BSI but higher for HA-BSI than rates obtained in a previous South African study conducted 20 years previously [6]. The isolation of 5.7% pathogens from blood culture specimens in the present study is similar to that from another referral hospital in Cape Town [7]. Possible reasons for the low pathogen yield in our setting may include inadequate volumes of blood inoculated into blood culture specimen bottles, lack of clear clinical indications for performing blood cultures potentially inflating the number of negative cultures and the prior use of antimicrobials, particularly ceftriaxone, as part of the syndromic case management of sick children at primary health care facilities [5, 9, 17]. The contaminant prevalence of 6.6% from 16,951 blood cultures is more than double the internationally acceptable contamination rate of 2–3%, suggesting that urgent attention is needed to improve aseptic measures when obtaining specimens for blood culture [18, 19]. A previous analysis of blood culture results over a 5-year period (2008–2012) from the same institution highlighted problems of high contamination rates particularly in the less than one-year age group and low pathogen yield, and also documented the predominance of gram-negative pathogens. [8].

*S. aureus, E.coli, K. pneumoniae* and *A. baumanii* predominated in our study. This spectrum of organisms is similar to that documented in other African studies [5–7]. Non-typhoidal *Salmonella* is a much more frequent cause of BSI in African countries to the north of South Africa, especially those in malaria-endemic areas, but has been reported as an important cause of BSI in an early South African study [20].

At the time of BSI, 27.5% of the episodes were not associated with a clinical focus of infection and 65.1% had fever (≥ 38 °C) or hypothermia (< 35.5 °C). A study done in Guinea-Bissau showed that the sensitivity of fever for detecting BSI was low at 54% with a positive predictive value (PPV) of 12% [21]. However, a Tanzanian study showed that temperature ≥ 38.5 °C was a strong predictor of BSI (odds ratio (OR) 7, 95% CI: 2.2–14.8, *P* = 0.0001) although the number of patients enrolled in that study was small [22]. Furthermore, a Malawian study of 225 hospitalised children found that the presence of at least one of five clinical features (oral thrush, malnutrition, chronic cough, lethargy on history and lethargy on examination) predicted BSI with a sensitivity of 69% [23].

Severe underweight was present in 25.2% of children in our study and was a significant predictor of 14-day mortality. These findings are in agreement with previous African studies showing that severe malnutrition significantly increases the risk of BSI, and that nutritional status is a significant determinant of survival in children with BSI [5, 24]. Although HIV infection was known to be present in 13.4% of the study cohort it did not influence 14-day mortality. Approximately 60% of the HIV-infected group was on ART at the time of BSI. This may partly explain why HIV infection did not influence mortality.

Our results show that ESBL-producing *K. pneumoniae* and *E. coli* have become important causes of CA- and HA-BSI. Of particular concern was that 30% of *E.coli* isolates in the CA-BSI group were ESBL-producing organisms. A recent study from another Cape Town hospital documented lower prevalence of CA-BSI caused by ESBL-producing *E.coli* (11.2%) and HA-BSI caused by ESBL-producing *K.pneumoniae* (78.3%) [7]. Although carbapenem-resistant *Enterobacteriaceae* infection was not observed during the study period, carbapenem over-utilisation as observed in the present study (data not shown) may with time lead to the emergence of carbapenem resistant BSI isolates.

### Study strengths and limitations

The study describes the spectrum of organisms causing BSI in children. The results obtained highlight the growing concern of antibiotic resistance developing particularly in CA–BSI. One limitation was the lack of susceptibility data for piperacillin-tazobactam due to technical limitations at the time of the study. With on-going surveillance of organisms causing BSI and their susceptibility patterns, empiric antimicrobial choices may need to be altered in the future. Subsequent to the study period, updated definitions of reporting laboratory confirmed BSI were release by the CDC [25]. These definitions were not utilised in the current study and as a result, few of the HA-BSI episodes may have been misclassified. Organisms implicated in mucosal barrier injury BSI were also not stratified.

Due to the retrospective study design, there are limitations in the completeness and availability of clinical and laboratory data. The true burden of community-acquired BSI may have been underestimated. Before sick children are referred from primary and secondary health facilities to our hospital, they are often administered broad-spectrum antibiotics. This practice is consistent with the World Health Organization Integrated Management of Childhood Illness guidelines that have been implemented in South Africa, and frequently results in no growth on blood culture [9]. Even though the study cohort included neonates, the results obtained would not be an accurate reflection of the burden of BSI in this special group of infants. We were only able to explore a limited number of potential risk factors of BSI mortality. Further studies with larger sample sizes are required to provide a more in depth understanding of this aspect of BSI. Furthermore, it was not possible to explore risk factors associated with the acquisition of a BSI due to the lack of a suitable control group of children without BSI.

## Conclusion

Our study provides insights about the risk, aetiology, clinical manifestations, antimicrobial therapy and outcomes of BSI in a sub-Saharan African setting characterized by high HIV prevalence. Further studies are required to gain a comprehensive understanding of the impact of BSI on the childhood population in SSA.

## Abbreviations

ART: Antiretroviral therapy
BSI: Bloodstream infection
CA: Community-acquired
CLSI: Clinical Laboratory standards institute
CRE: Carbapenem-resistant *Enterobacteriacae*
CRP: C-reactive protein
ESBL: Extended-spectrum beta lactamase
FBC: Full blood count
HA: Hospital-acquired
HCA: Healthcare-associated
HIV infection: Human immunodeficiency virus infection
IQR: Interquartile range
NHLS: National health laboratory service
PCT: Procalcitonin
SSA: Sub-Saharan Africa
UWFA: Underweight for age
WAZ: Weight for age z-score
WCC: White cell count
